# Supplementary figures and images for: Salmonella manipulates the host to drive pathogenicity via induction of interleukin 1β
Source: PLoS Biol. 2024 Jan 18;22(1):e3002486. doi: 10.1371/journal.pbio.3002486 (PMC10826948; doi:10.1371/journal.pbio.3002486)

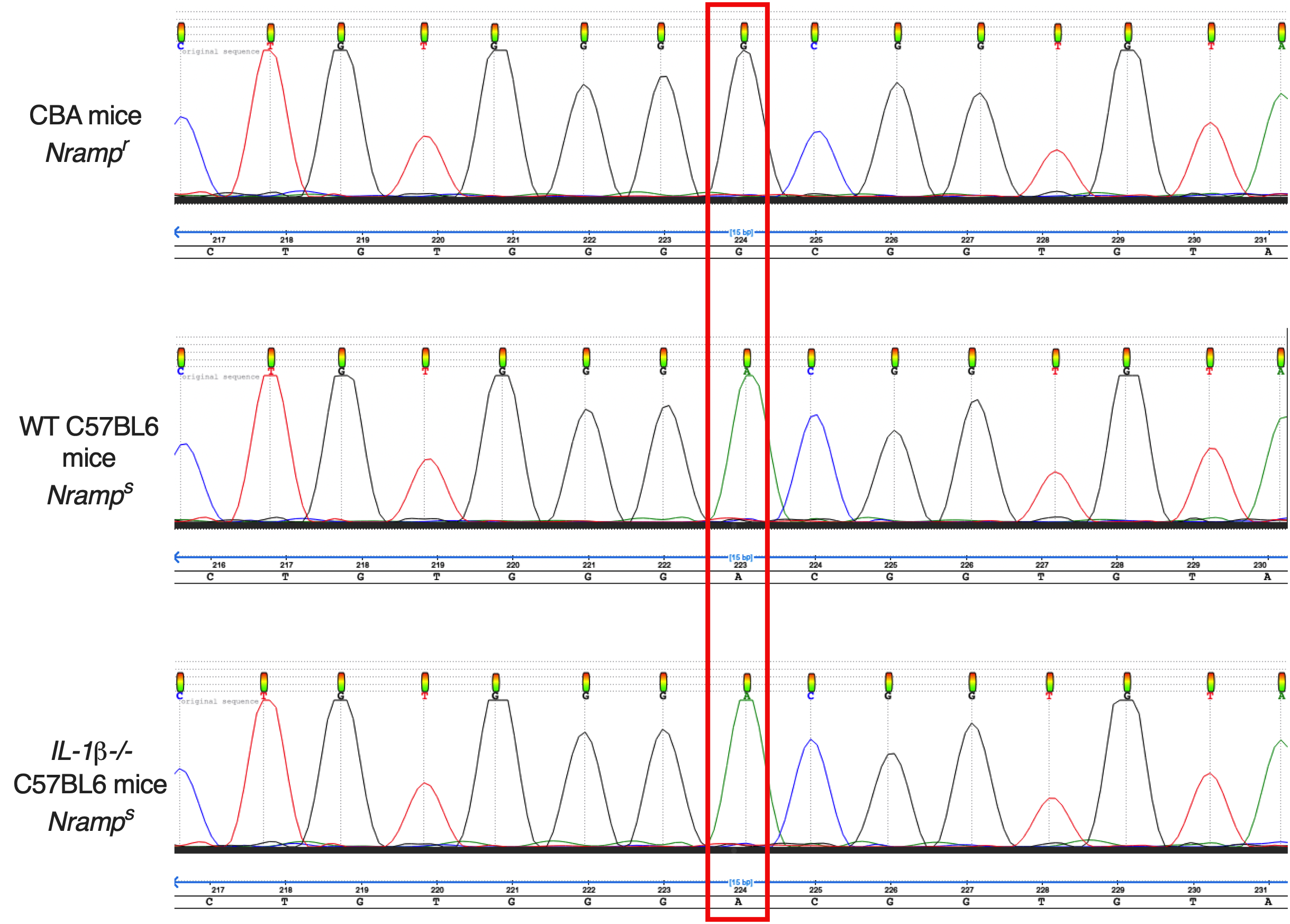

Supplement: S1 Fig — Sequencing of genomic DNA from CBA mice (Nrampr), WT C57BL/6 mice (NrampS), and and IL-1β -/- mice on a C57BL/6 background (NrampS). The red rectangle highlights the sensitivity mutation G->A. (TIFF) [file pbio.3002486.s001.tiff]

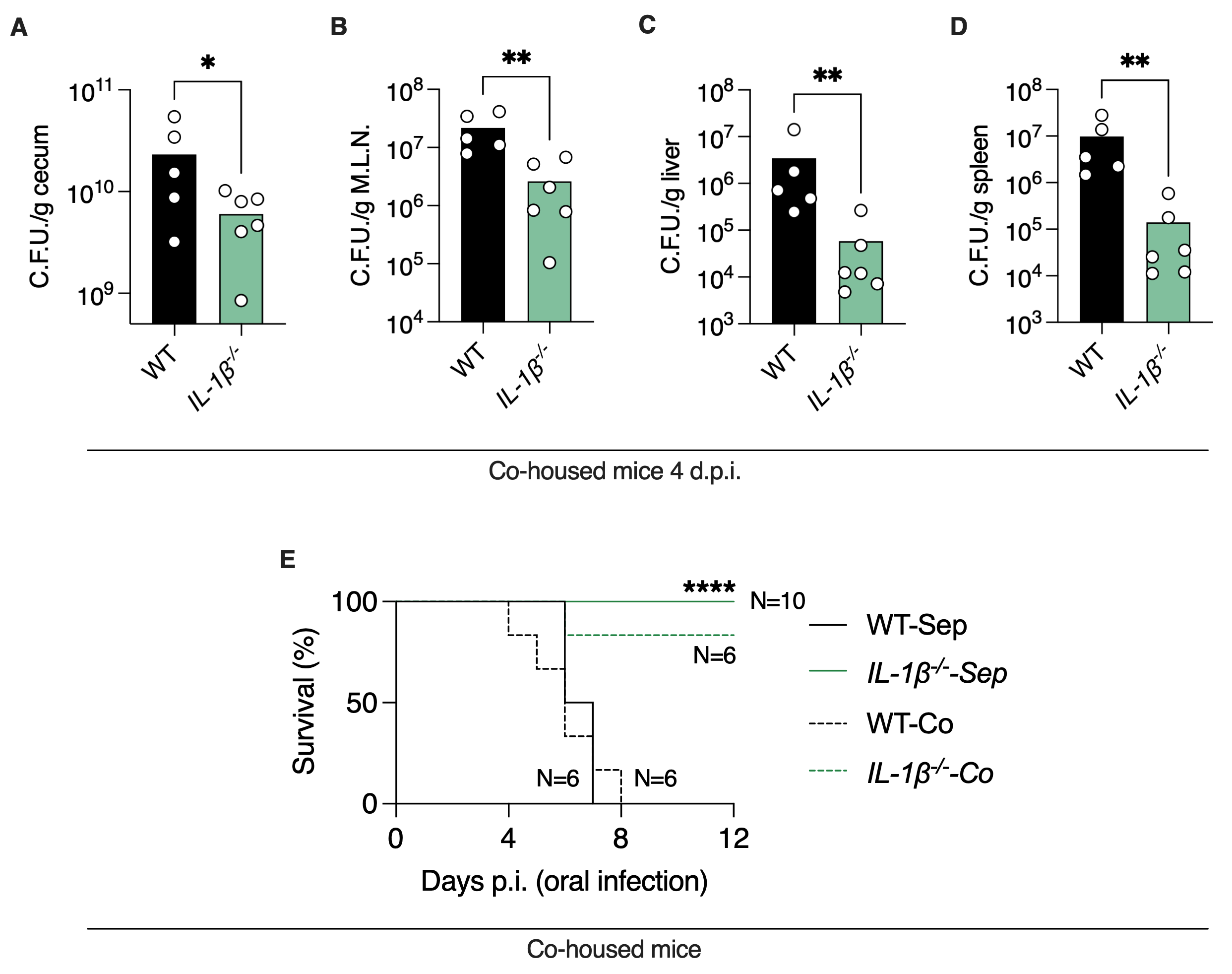

Supplement: S2 Fig — (A–D) Salmonella C.F.U. 4 days post-infection in cecal content (A), M.L.N. (B), liver (C), and spleen (D) of co-housed mice infected with 107 C.F.U. Salmonella enterica serovar typhimurium (SL1334) 24 h after pretreatment with 20 mg streptomycin. (E) Survival percentage of mice infected orally with Salmonella. (A–D) Each dot represents a mouse. These data are representative of 1 experiment. *P < 0.05; **P < 0.01; ****P < 0.0001. (A–D) Mann–Whitney test. (E) Mantel–Cox test. C.F.U., colony-forming units; Sep, separately housed; Co, co-housed; d.p.i., days post-infection. Numerical values are in S1 Data. (TIFF) [file pbio.3002486.s002.tiff]

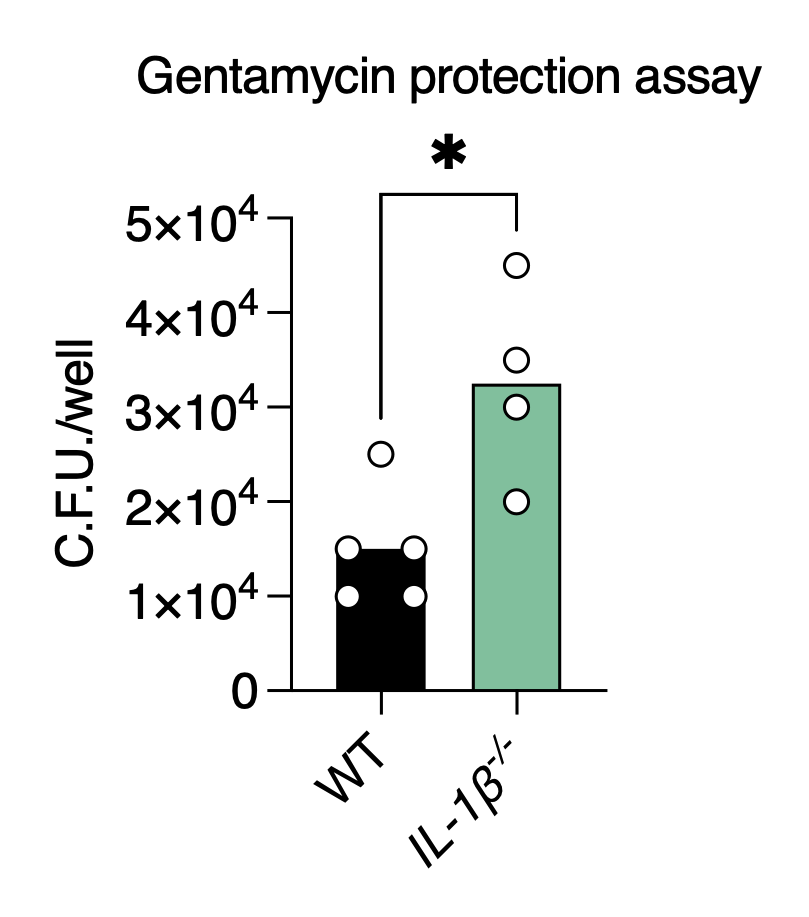

Supplement: S3 Fig — (A) Gentamycin protection assay using peritoneal macrophages extracted from mice infected with Salmonella ex vivo with a MOI of 3. These data are representative of 1 experiment. *P < 0.05; Student’s t test. Numerical values are in S1 Data. (TIFF) [file pbio.3002486.s003.tiff]

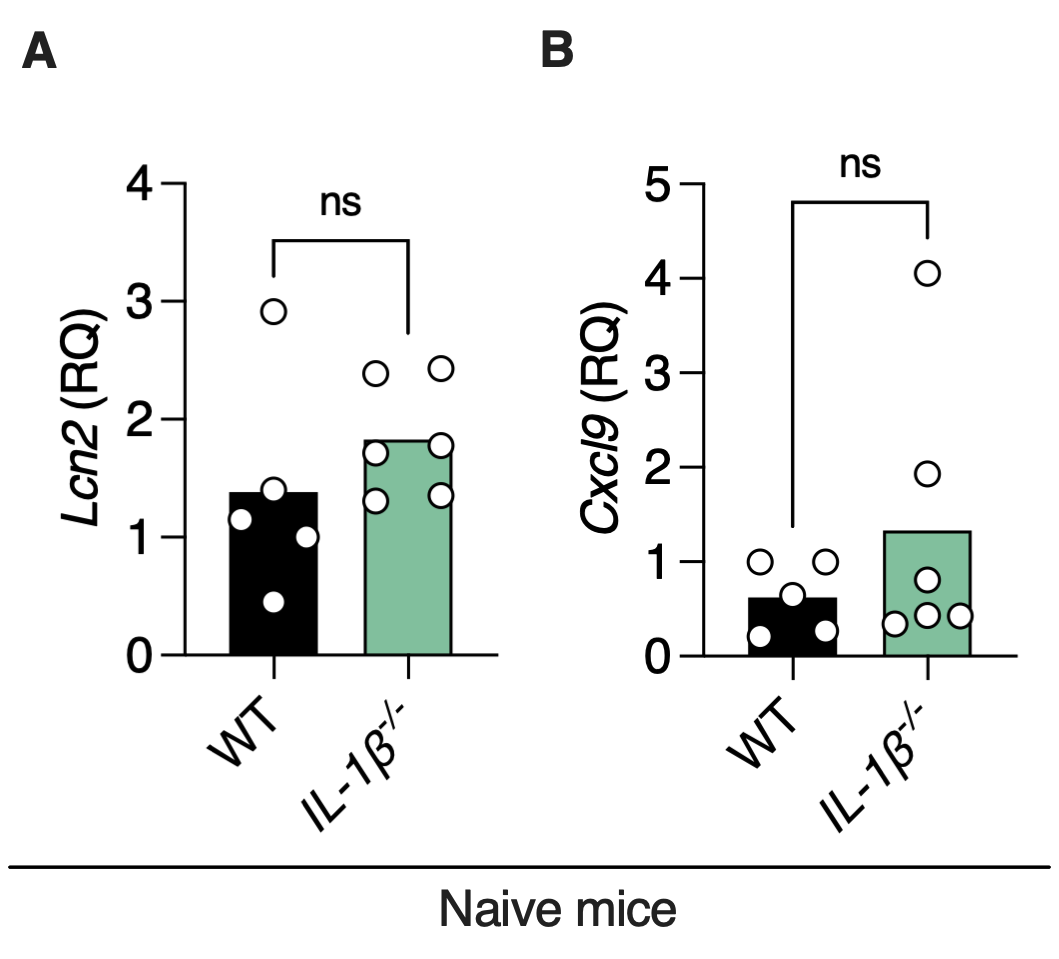

Supplement: S4 Fig — (A and B) qPCR analysis of (A) Lcn2 and (B) Cxcl9 transcripts in colons of naïve mice. Expression was normalized to 18S. These data are representative of 1 experiment. ns, not statistically significant; RQ, relative quantity. Student’s t test. Numerical values are in S1 Data. (TIFF) [file pbio.3002486.s004.tiff]

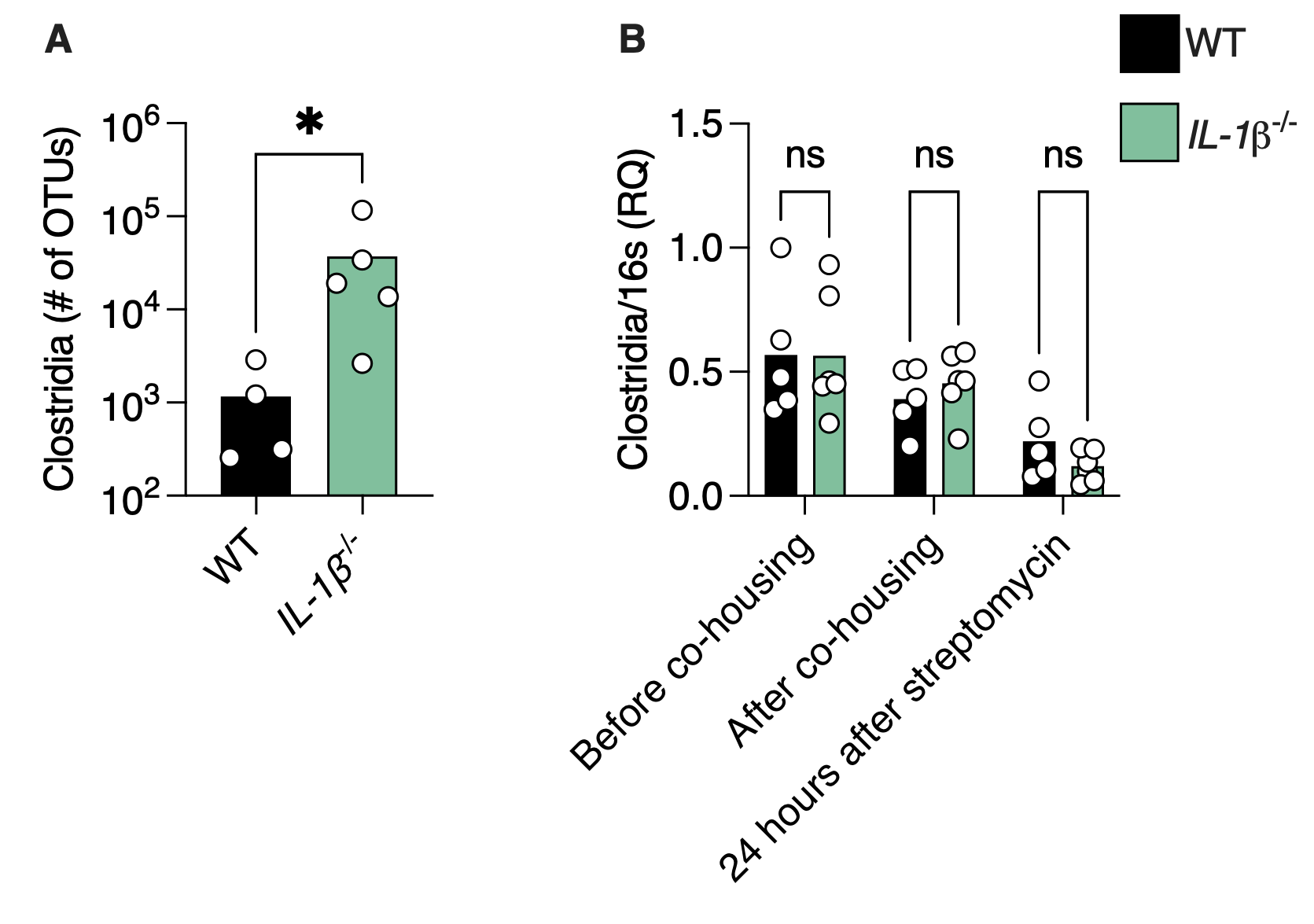

Supplement: S5 Fig — (A) Absolute number of Clostridia OTUs in gut microbiota of Salmonella-infected mice as in Fig 3. (B) qPCR analysis of levels of the class Clostridia in uninfected mice housed and treated as indicated. Each dot represents a mouse. These data are representative of 1 experiment. *P < 0.05. Student’s t test. OTUs, operational taxonomic unit; RQ, relative quantity. Numerical values are in S1 Data. The underlying data for this figure can be found at GSE252071. (TIFF) [file pbio.3002486.s005.tiff]

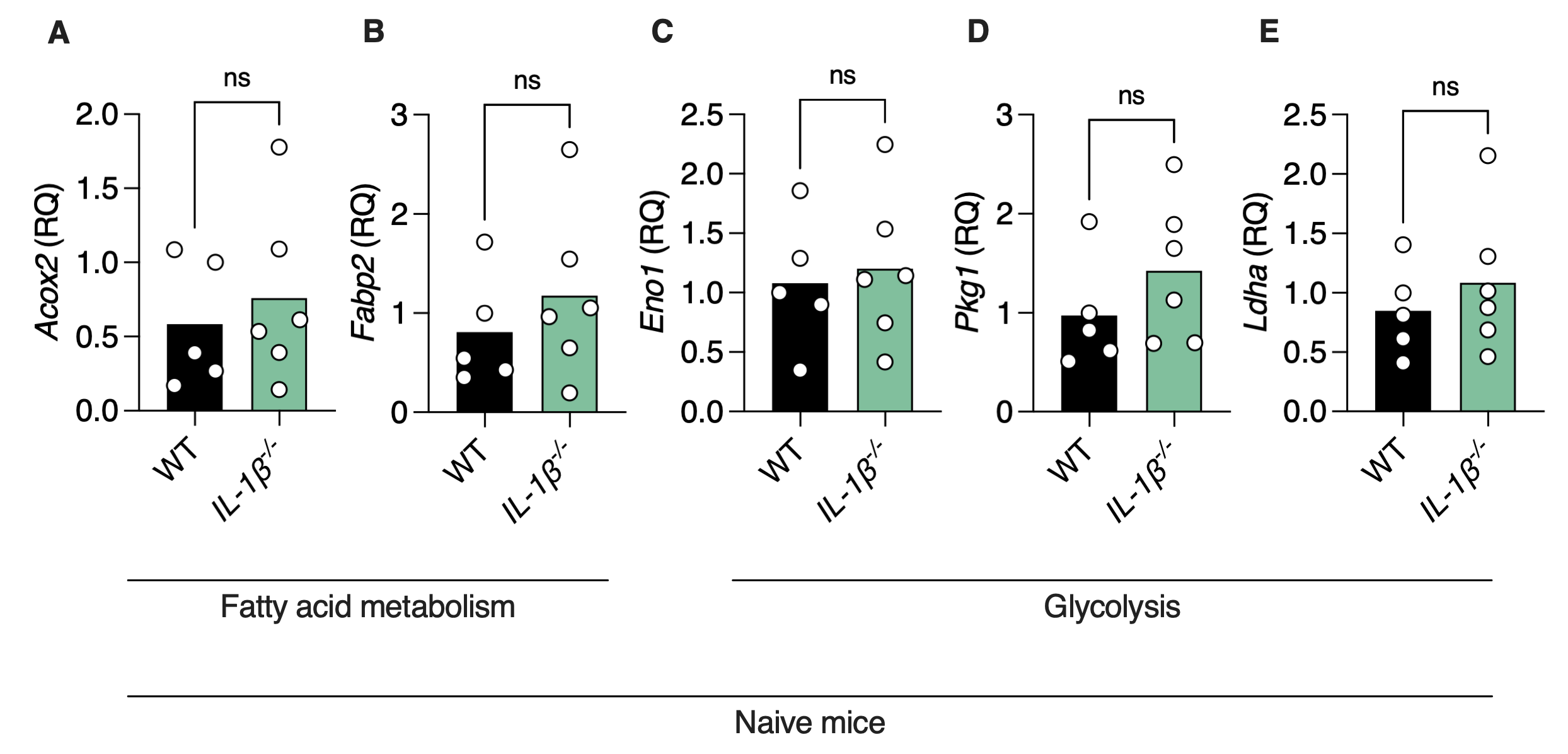

Supplement: S6 Fig — qPCR analysis of (A and B) transcripts involved in fatty acid metabolism and (C–E) transcripts involved in glycolysis in colons of naïve mice. Expression was normalized to 18S. These data are representative of 1 experiment. ns, not statistically significant; RQ, relative quantity. Student’s t test. Numerical values are in S1 Data. (TIFF) [file pbio.3002486.s006.tiff]

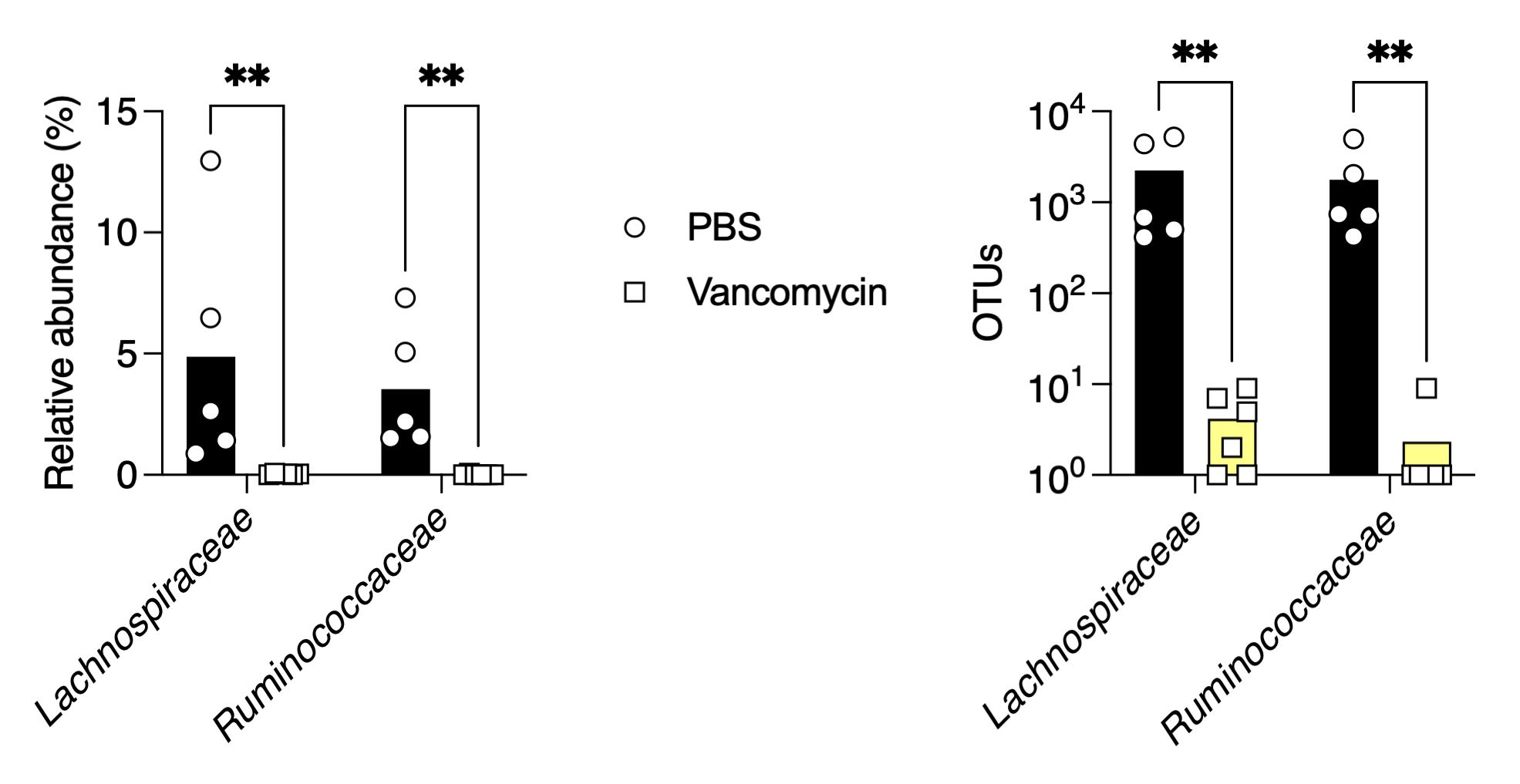

Supplement: S7 Fig — 16S rRNA sequencing was performed to characterize gut microbiota composition of mice treated with vancomycin for 3 days. Relative abundance (left) and absolute OTU reads (right) of SCFA-producing members of the Clostridia class at the family level. Each symbol represents a mouse. OTU, operational taxonomic unit. These data are representative of 1 experiment. **P < 0.01; Student’s t test. Numerical values are in S1 Data. (TIFF) [file pbio.3002486.s007.tiff]

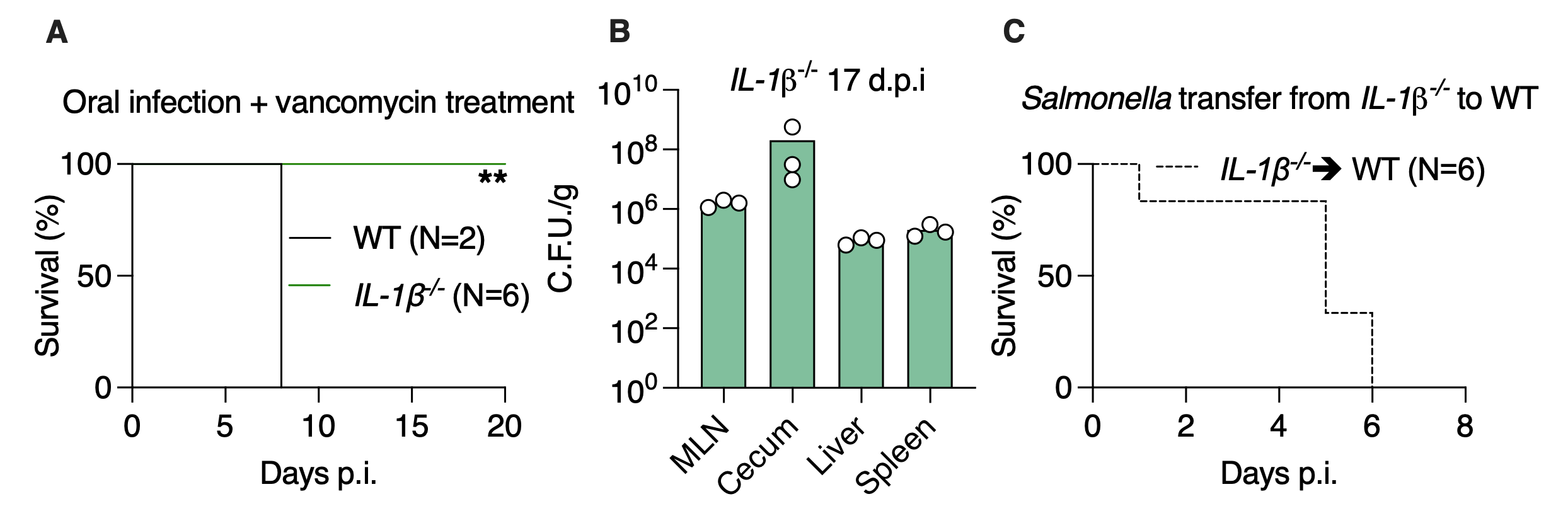

Supplement: S8 Fig — (A) Survival of vancomycin-treated mice infected orally. (B) Salmonella C.F.U. in the indicated organs of IL-1β -/- mice 17 d.p.i. Each dot represents a mouse. (C) Survival of WT mice infected with Salmonella isolated from IL-1β -/- mice 21 d.p.i. **P < 0.01. (A) Mantel–Cox test. These data are representative of 1 experiment. d.p.i., days post-infection. Numerical values are in S1 Data. (TIFF) [file pbio.3002486.s008.tiff]

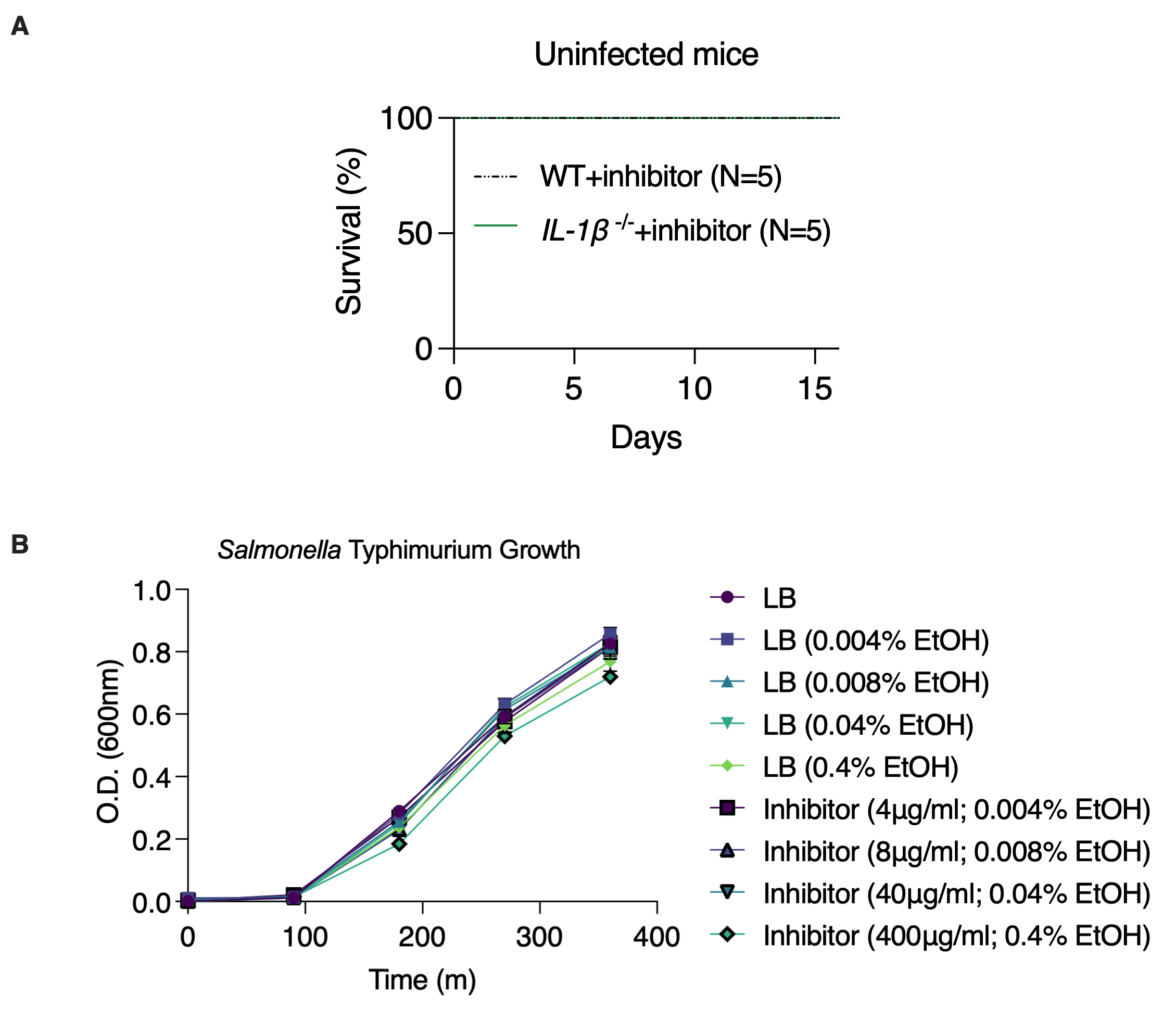

Supplement: S9 Fig — (A) Survival of uninfected mice treated as in Fig 5. (B) Growth curve of Salmonella treated as indicated. These data are representative of 1 experiment. Numerical values are in S1 Data. (TIFF) [file pbio.3002486.s009.tiff]
